# Pilot implementation of the GAIHN point prevalence survey on healthcare-associated infections at a Jordanian tertiary oncology hospital, an international partnership with the U.S. Centers for Disease Control and Prevention

**DOI:** 10.1017/ash.2026.10859

**Published:** 2026-09-23

**Authors:** Sawsan Mubarak, Joud Jarrah, Manar Makhlouf, Rawan Hajir, Sulafa Al Ansari, Hebah Beides, Hadeel AlGhawrie

**Affiliations:** King Hussein Cancer Center, Jordan

## Abstract

**Background:**

The Global Action in Healthcare Network develops point prevalence survey (PPS) protocol for healthcare-associated infection surveillance, particularly in resource-limited settings. This study reports the first field implementation of the Global Action in Healthcare Network (GAIHN) HAI PPS protocol and aims to evaluate the protocol and to estimate healthcare-associated infection (HAI) prevalence and antimicrobial use at a tertiary oncology hospital in Jordan.

**Methods:**

Descriptive, cross-sectional point prevalence survey conducted using a standardized GAIHN HAI PPS protocol, coordinated by the U.S. Centers for Disease Control and Prevention, was implemented using an algorithmic REDCap-based data collection system. Targeted HAIs included bloodstream infection, pneumonia, urinary tract infection, and surgical site infection. Antimicrobial use, device exposure, microbiology, and ward-level characteristics were recorded. HAI prevalence was calculated with 95% confidence intervals.

**Results:**

A total of 124 patients were surveyed, 73 (58.9%) underwent full chart review. Antimicrobial use was documented in 63.7% of surveyed patients, with an Access-to-Watch ratio of 0.23. Eight HAI events were identified in six patients, corresponding to an overall HAI prevalence of 4.8% (6/124; 95% CI 2.2–10.1). Pneumonia was the most frequent HAI type. Gram-negative organisms accounted for 60% of isolates. Patients in intensive care was more likely to meet criteria for detailed review.

**Conclusion:**

This pilot demonstrated feasibility for the GAIHN HAI PPS methodology and data collection system. It offers a framework for improving IPC programs and implementing evidence-based practices that can be adapted and scaled across the region, contributing to the global effort to reduce HAIs and combat antimicrobial resistance.

## Background

Healthcare-associated infections (HAIs) are defined as infections not present at admission that develop during the course of receiving healthcare treatment (≥48 h after hospital admission or within a defined period after a procedure or discharge).^1,2^ HAIs cause significant morbidity and mortality and account for billions of dollars in healthcare costs globally. While the majority of HAIs are preventable through effective infection prevention and control (IPC), robust surveillance data are critical to informing and measuring the impact of IPC efforts.^3–5^

The burden of HAIs is disproportionately high in low- and middle-income countries, where gaps in IPC infrastructure, antimicrobial stewardship, and surveillance capacity persist, and risk is particularly elevated in intensive care units and other high-complexity settings such as tertiary oncology centers, where immunocompromised patient populations, frequent invasive procedures, and substantial antimicrobial exposure heighten infection risk.^4,6–9^

A multi-country prevalence surveys of HAIs in the European Union and European Economic Area (EU/EEA) showed a prevalence of 5.9% in 2016–2017, which increased to 7.1% in the third ECDC point prevalence survey conducted in 2022–2023.^11,12^ HAI prevalence was found to be 3.2% among patients in 199 hospitals in the United States of America (USA) in 2015.^13^ High-complexity settings such as tertiary oncology centers represent additional priority environments for surveillance due to increased risk of HAIs given immunocompromised patient populations, frequent invasive procedures, and substantial antimicrobial exposure.

In the Middle East region, research studies report various HAI rates caused by a wide range of microorganisms, with various patterns of antimicrobial resistance.^14–17^ The first point-prevalence survey in the Middle East was conducted in 2018, including seven countries: Egypt, the Kingdom of Saudi Arabia, the United Arab Emirates, Lebanon, Oman, Kuwait, and Bahrain. This study revealed that the overall HAIs was 11.2%, which indicates that the point prevalence of HAI is high in a sample of Middle Eastern countries. Additionally, there were high levels of resistance to antimicrobials among *Acinetobacter baumannii*, *Enterobacter cloacae*, *Klebsiella pneumoniae*, and other *Klebsiella* species.^15^ A systematic review encompassing 25 years of HAI data from Middle Eastern countries confirmed similarly elevated pooled estimates, underscoring the persistent and underappreciated regional burden.^17^

The Global Action in Healthcare Network (GAIHN) was launched in 2021 as part of the U.S. Centers for Disease Control and Prevention’s (CDC’s) Global Antimicrobial Resistance Laboratory and Response Network. GAIHN is a global collaborative network consisting of countries, institutions, and partners at global, regional, national, and subnational levels working together to address HAIs and antimicrobial resistance in healthcare settings. GAIHN established functional networks in four countries: Brazil, Indonesia, Jordan, and the Philippines. Recognizing the potential of pragmatic surveillance approaches, the U.S. Centers for Disease Control and Prevention (CDC) launched GAIHN in 2021 to strengthen global efforts to prevent and control HAIs and antimicrobial resistance across resource-variable settings.^10^

Point prevalence surveys (PPS) offer a practical alternative to more resource-intensive surveillance methods. Established PPS frameworks including those developed by the World Health Organization and the European Centre for Disease Prevention and Control have demonstrated the value of standardized, time-limited assessments of HAI burden and antimicrobial use across diverse healthcare settings.^18^ Building on this foundation, the GAIHN HAI PPS represents a further adaptation specifically designed for rapid deployment in resource-variable environments: it is digitally enabled through an algorithmic REDCap-based data collection system, structured to minimize staff burden through parsimonious data entry, and explicitly oriented toward local capacity building.^10^

The implementation of the HAI PPS at King Hussein Cancer Center (KHCC), a large tertiary oncology center serving patients from Jordan and the surrounding region, represented the first field application of the GAIHN HAI PPS protocol. As a high-complexity facility caring for immunocompromised patients with substantial device utilization and antimicrobial exposure, KHCC offered a particularly demanding and informative environment in which to test structured HAI surveillance. This pilot aimed not only to generate local estimates of HAI prevalence and antimicrobial use, but to serve as real-world systems test of the GAIHN protocol: evaluating whether a standardized, algorithmically guided survey could be executed by a local IPC team, yield reliable epidemiologic data, and produce actionable findings within the operational constraints of a busy tertiary hospital in a middle-income country.

## Methods

### Survey setting and design

The survey was conducted at KHCC in Amman, Jordan, during June and August 2023. KHCC is a tertiary oncology referral center providing specialized oncology care. The Infection Prevention and Control program included five full-time IPC professionals, supported by an antimicrobial stewardship team of three full-time consultants and an active multidisciplinary IPC committee.

The survey followed the standardized PPS protocol developed through the GAIHN coordinated by the U.S. Centers for Disease Control and Prevention.^10^ The protocol provides standardized case definitions, data collection instruments, and analytic guidance for estimating HAI prevalence and antimicrobial use.

### Ward and patient eligibility

Wards were eligible if they admitted adult patients (≥18 yr) for more than 24 hours. Final ward selection was determined by the facility survey team. Nine wards were included: two ICUs, two surgical wards, and five medical wards. Final ward selection was determined by the facility survey team to ensure representation of major clinical service types, including medical, surgical, and intensive care units, in proportion to their contribution to overall bed capacity. Wards were also selected based on operational feasibility, including anticipated patient volume, staff availability to support data collection, and the ability to complete chart review within the three-day window allotted per ward. Wards excluded from the eligible pool (e.g., pediatric, day-procedure, and outpatient infusion units) were not considered for selection, consistent with the GAIHN protocol’s focus on adult inpatient settings.

Patients were eligible if they were ≥18 years old and were admitted for 72 hours or more and present on the ward at or before 8:00 a.m. on the survey date. Data collection for each ward was completed within three days of its survey date. Eligibility for full chart review was determined algorithmically through the REDCap-based data system rather than individual surveyor judgment. Patients were flagged for full chart review if they met at least one of two criteria: receipt of systemic antimicrobial therapy on the survey date or a documented surgical procedure within the preceding 30 days. Patients meeting neither criterion were excluded from full chart review, in keeping with the GAIHN protocol’s focus on patients at highest risk for HAI.

### Case definitions and classification

HAI case definitions were applied in accordance with the standardized GAIHN HAI PPS protocol. Targeted infections included pneumonia, urinary tract infection, bloodstream infection, and surgical site infection. Infections meeting surveillance criteria were classified as healthcare-associated based on timing relative to admission. Infections not meeting HAI criteria were categorized as not associated with the current admission. Consistent with the pilot’s design as a single-day, inpatient point prevalence survey, postdischarge case finding for surgical site infection was not performed. SSI ascertainment was therefore limited to infections identified during the inpatient stay among patients present on surveyed wards on the survey date. A detailed summary of these definitions is provided in Supplemental Material.

### Data collection procedures

The KHCC IPC team conducted the survey following structured training on case definitions, chart abstraction, and data entry procedures prior to implementation. Chart review was performed using the KHCC electronic medical record system, which includes patient demographics, current antimicrobial therapy, surgical history, signs and symptoms of infection, relevant laboratory and radiologic findings, and presence of invasive devices. Data abstraction focused on the 14 days preceding survey date, except for prior surgical procedures where earlier documentation during hospitalization was reviewed.

Antimicrobials were classified according to the WHO Access, Watch, and Reserve (AWaRe) framework, which categorizes antimicrobial agents based on their spectrum of activity and resistance potential. ‘Access’ antibiotics are first- or second-choice agents with a narrower spectrum and lower resistance potential; ‘Watch’ antibiotics have higher resistance potential and are prioritized as key targets of stewardship programs; ‘Reserve’ antibiotics are last-resort agents reserved for confirmed or suspected multidrug-resistant infections. The Access-to-Watch ratio was calculated as the number of patients receiving Access-category agents divided by the number receiving Watch-category agents.

Data were collected on three invasive devices commonly associated with specific HAI: central venous catheters (CVCs), indwelling urinary catheters, and mechanical ventilation. Consistent with the GAIHN-HAI PPS data collection instrument, device presence was recorded if the device was in place at the date of the survey or at any point in the 14 days prior to the survey date. Device days were calculated as the total number of documented days each device was present during this time frame. Facility-level characteristics, including bed capacity, staffing composition, and ward types, were also recorded.

Data were collected using REDCap-based electronic forms.^19^ The REDCap GAIHN HAI PPS data collection system is algorithm-based which allows for parsimonious data entry to collect only pertinent information of patients at risk for HAIs. Initial screening for detailed HAI assessment was based on antimicrobial therapy status on the survey date and previous surgery. Patients receiving antimicrobial therapy and/or with previous surgery underwent detailed review to determine whether they met standardized surveillance case definitions for targeted infections.

### Statistical analysis

Point prevalence estimates and corresponding 95 percent confidence intervals were calculated using the Wilson score method. Analyses were conducted using R version 4.4.0 with the binom package for binomial confidence interval estimation.

## Results

### Facility characteristics

KHCC is a 360-bed tertiary oncology hospital, including 35 ICU beds. In 2022, the hospital reported 17,402 admissions and 101,383 patient-days. The microbiology laboratory processed 23,892 blood culture sets during the same year.

### Patient population

A total of 124 adult inpatients were included across two ICUs, two surgical wards, and five medial wards. The median age of surveyed patients was 54 years. A subset of 73 patients (46%; 73/124) met full chart review criteria for HAI consideration. Patient demographics by indication for full chart review is illustrated in Table 1.

**Table 1.** Patient demographics and clinical information by indication for full chart review—KHCC HAI PPS Table 1 long description.

	Total (N = 124)	Full review indicated (N = 73)	Full review not indicated (N = 51)
Median age (IQR)	54 (16)	52 (14)	55 (17.5)
Sex			
Male (%)	63 (51%)	34 (47%)	29 (57%)
Female (%)	61 (49%)	39 (53%)	22 (43%)
Antimicrobial on survey date			
Yes (%)	79 (64%)	66 (90%)	13 (25%)
No (%)	45 (36%)	7 (10%)	38 (75%)
Surgery in the 30 days prior to or during admission			
Yes (%)	31 (25%)	31 (42%)	0
No (%)	93 (75%)	42 (58%)	51 (100%)
Median length of stay (Days)	5 (0–58)	7 (1–58)	3 (0–41)
Ward type			
Medical (non-ICU) (%)	83 (67%)	41 (56%)	42 (82%)
Surgical (non-ICU) (%)	32 (26%)	25 (34%)	7 (14%)
ICU (%)	9 (7%)	7 (10%)	2 (4%)

### Patient screening

Seventy-three patients (58.9%) met criteria for full chart review. Of the total patients undergoing full chart review, 66 patients were receiving systemic antimicrobial therapy on the survey date and 31 patients had documentation of a surgical procedure within the preceding 30 days. Some patients met both criteria. Eligibility for full review was more common among ICU and postsurgical patients.

Of the 73 patients who underwent full chart review, central venous catheters were present in 13 patients (17.8%), with a total of 519 device-days and an average duration of 28.8 days per patient. Indwelling urinary catheters were the most frequently observed device, documented in 22 patients (30.1%), accounting for 190 device-days and an average of 8 days per patient. Endotracheal intubation was less prevalent, recorded in 3 patients (4.1%), with 51 total device-days and an average duration of 8.5 days per patient.

### Antimicrobial use

Among all surveyed patient, seventy-nine patients (63.7%) were receiving at least one systemic antimicrobial agent on the survey date. Among these, 65 patients (82.3%) were receiving at least one antimicrobial with a documented indication of infection treatment, while 57.0% (45/79) were receiving multiple antimicrobials (range 1–5 antimicrobials). Based on WHO AWaRe classification,^15^ 77% were categorized as Watch, 18% as Access, and 5% as Reserve, yielding an Access-to-Watch ratio of 0.23. The distribution of prescribed antimicrobials by WHO anatomical therapeutic chemical (ATC) classification is summarized in Table 2.

**Table 2.** Summary of prescribed antimicrobials by WHO anatomical therapeutic chemical classification—KHCC HAI PPS Table 2 long description.

Antimicrobial medication	Number of patients receiving (%)
Total patients on an antimicrobial	79 (100)
Beta-lactam antibacterials, penicillins	
Piperacillin and beta-lactamase inhibitor	26 (32.9)
Amoxicillin and beta-lactamase inhibitor	2 (2.5)
Other beta-lactam antibacterials	
Meropenem	20 (25.3)
Cefazolin	12 (15.2)
Ceftriaxone	9 (11.4)
Cefepime	6 (7.6)
Ceftazidime and beta-lactamase inhibitor	2 (2.5)
Aztreonam	1 (1.3)
Sulfonamides and trimethoprim	
Sulfamethoxazole and trimethoprim	3 (3.8)
Aminoglycoside antibacterials	
Amikacin	1 (1.3)
Quinolone antibacterials	
Ciprofloxacin	7 (8.9)
Levofloxacin	3 (3.8)
Other antibacterials	
Vancomycin	30 (38.0)
Metronidazole	6 (7.6)
Colistin	4 (5.1)
Systemic antimycotics	
Isavuconazole	3 (3.8)
Micafungin	3 (3.8)
Voriconazole	3 (3.8)
Amphotericin B	2 (2.5)
Fluconazole	2 (2.5)
Direct-acting antivirals	
Acyclovir	3 (3.8)

### Microbiology

Of the 73 patients meeting criteria for full chart review, 67.1% (49/73) had at least one culture ordered and 24.7% (18/73) had at least one positive culture result. A total of 164 cultures were obtained, of which 18.3% were positive. A total of 84 blood cultures, 54 urine cultures, 16 respiratory cultures, and 10 wound/abscess cultures were obtained. Culture positivity was highest among respiratory cultures (56.3%; 9/16) and lowest among blood cultures (9.5%; 8/84). In total, 40 organisms were isolated. Twenty percent of the isolates were gram-positive, 60% were gram-negative, and 20% were fungi.

### *Healthcare*-*associated infections*

Eight infections involving 6 patients met standardized surveillance case definitions for healthcare-associated infection. The median interval from admission to infection was 7 days (range 4–16 d). Among identified HAIs, there was one urinary tract infection, two bloodstream infections, three pneumonias, and two surgical site infections. Overall, HAI prevalence for all HAIs in the wards surveyed was 4.8% (6 of 124 patients). The combined prevalence of UTI, BSI, and pneumonia was 3.2% (4/124); SSI prevalence among patients who underwent recent surgery was 6.5% (2/31). Full details by patient, relevant device presence, and ward are presented in Table 3.

**Table 3.** List of eight infections classified as healthcare-associated by patient, presence of a relevant device, and ward surveyed—KHCC HAI PPS Table 3 long description.

	HAI	Relevant device	Ward
Patient A	Bloodstream infection	Yes	Surgical
Patient B	Bloodstream infection	No	Medical
Patient B	Pneumonia	No	Medical
Patient C	Pneumonia	Yes	ICU
Patient D	Pneumonia	Yes	ICU
Patient D	Urinary tract infection	Yes	ICU
Patient E	Surgical Site Infection	N/A	Medical/Surgical
Patient F	Surgical Site Infection	N/A	Surgical

## Discussion

This survey represents the first implementation of the GAIHN HAI point prevalence survey protocol. Conducted at the KHCC, a tertiary oncology hospital, the survey generated baseline estimates of HAIs and antimicrobial use while providing practical insight into the feasibility of conducting structured HAI surveillance in a high-complexity facility in a middle-income country.

As a specialized oncology facility caring for immunocompromised patients, KHCC represents a distinct clinical environment in which infection risk and antimicrobial exposure patterns may differ from those of general acute care hospitals. At the same time, KHCC maintains an exceptional IPC infrastructure that is uncommon in the regional context with a dedicated full-time IPC team, an active antimicrobial stewardship program, a multidisciplinary IPC committee, and sustained engagement with international surveillance networks. This combination of elevated baseline infection risk and robust preventive capacity makes KHCC a particularly informative setting for piloting the GAIHN HAI PPS methodology, as findings reflect both the clinical complexity of oncology care and the operational feasibility of implementing standardized surveillance within a well-resourced regional institution.

The overall HAI point prevalence in the wards surveyed of 4.8% (95% CI: 2.2%–10.1%) falls within the lower range of estimates reported in regional and international PPS studies. A multi-country point-prevalence survey conducted in seven Middle Eastern countries reported a prevalence of 11.2% in acute care settings.^20^ Pooled HAI prevalence in mixed patient populations has been reported at 7.6% in high-income countries, while hospitalwide prevalence in low- and middle-income countries ranged from 5.7% to 19.1%, with a pooled prevalence of 10.1%, and was notably higher in high- than in low-quality studies (15.5% vs 8.5%).^8^ Differences across studies likely reflect variation in patient populations, case mix, surveillance methodology, diagnostic capacity, and healthcare infrastructure.

Antimicrobial use was common, with 63.7% receiving at least one systemic agent and more than half of treated patients receiving combination therapy. WHO recommends that at least 60% of a country’s or facility’s total antimicrobial consumption come from the Access category, corresponding to a target Access-to-Watch ratio of at least 1.5; higher ratios indicate prescribing patterns weighted toward narrower-spectrum, lower-resistance-risk agents.^21^ The predominance of Watch-category antibiotics and the low Access-to-Watch ratio in our study was 0.23—which is below the WHO-recommended benchmark ratio of 1.5—indicate that prescribing at KHCC was heavily weighted toward broader-spectrum, higher-resistance-risk agents rather than first-line Access agents, and that’s highlight opportunities for antimicrobial stewardship review. These findings demonstrate the value of PPS methodology in generating standardized prescribing metrics aligned with WHO AWaRe benchmarks, providing actional data to inform local stewardship discussions.

From an implementation perspective, this pilot demonstrated that a structured PPS could be conducted within an established IPC framework using electronic medical records and trained IPC/nursing staff. The GAIHN HAI PPS data collection system was well-received and easy to use. The algorithmic guide to data entry led to parsimonious data collection, reducing staff burden. The system was able to identify HAIs in a standardized objective fashion. Staff participation in the survey improved IPC capacity and use of standardized case definitions and structured data abstraction, contributing to internal capacity building. At the same time, the activity required dedicated staff time and coordination across wards, underscoring the importance of aligning surveillance activities with available human resources. Sequential ward-level data collection extended the survey period and required careful tracking to avoid duplication. As in many PPS implementations, the completeness and accuracy of findings were dependent on the quality of clinical documentation. The insights gained from this pilot directly informed refinements to the global HAI PPS protocol and demonstrate its potential utility in diverse healthcare settings. As such, this experience represents an important step toward establishing sustainable, scalable HAI surveillance systems that support infection prevention and control across the full spectrum of resource availability.

In total, the survey was conducted by five members of the KHCC IPC team over approximately one month, with data extraction independently validated by a second reviewer for each patient chart, involving both KHCC-based and CDC/CRDF Global-based team members. This dual-review process supported data quality and consistency in the application of standardized case definitions. Findings from this pilot were used to directly inform infection prevention and control efforts at KHCC.

Several limitations merit consideration. The survey was conducted in a single tertiary oncology facility limiting generalizability. The sample size was modest, reducing precision for less common infection types. Data collection occurred during a defined period and may not reflect seasonal variation. In addition, detailed HAI assessment was limited to patients receiving systemic antimicrobial therapy or with recent surgery, consistent with the operational screening approach used to reduce staff burden. While pragmatic, this strategy may have resulted in under-ascertainment of untreated infections. Additionally, this pilot did not incorporate postdischarge case finding for SSI, as recommended by the GAIHN HAI PPS protocol. Because a substantial proportion of SSIs, particularly superficial incisional infections, are diagnosed after hospital discharge, their absence from this survey’s ascertainment method likely resulted in underestimation of true SSI prevalence and may partly explain the relatively low burden of this infection type observed here. Finally, reliance on electronic medical record documentation may have influenced case classification where clinical details were incomplete.

## Conclusion

This pilot implementation of the GAIHN-HAI Point Prevalence Survey protocol at King Hussein Cancer Center demonstrated the feasibility of using standardized, pragmatic surveillance methods to assess HAIs and antimicrobial use in a specialized care setting. The protocol enabled structured data collection across multiple domains relevant to IPC, including infection prevalence, antimicrobial prescribing, microbiologic testing, and device use.

In summary, this pilot demonstrated the feasibility of implementing the standardized GAIHN HAI point prevalence survey at a tertiary oncology hospital. The point prevalence survey generated useful baseline estimates of healthcare-associated infections and antimicrobial use in the wards surveyed and provided actionable insights into antimicrobial prescribing, device utilization, and infection burden. This pilot implementation supports the use of this pragmatic point prevalence survey approach to strengthen HAI surveillance and antimicrobial stewardship in specialized hospitals in resource-variable settings.

## Supporting information

10.1017/ash.2026.10859.sm001 Mubarak et al. supplementary material 1 Mubarak et al. supplementary material

10.1017/ash.2026.10859.sm002 Mubarak et al. supplementary material 2 Mubarak et al. supplementary material

10.1017/ash.2026.10859.sm003 Mubarak et al. supplementary material 3 Mubarak et al. supplementary material

10.1017/ash.2026.10859.sm004 Mubarak et al. supplementary material 4 Mubarak et al. supplementary material

## Data Availability

The datasets generated and analyzed during the current study are available from the corresponding author upon reasonable request.

## Supplementary material

The supplementary material for this article can be found at https://doi.org/10.1017/ash.2026.10859.

## Table 1. Long description

A table with 124 rows and 10 columns comparing patient demographics and clinical information by indication for full chart review. The columns are Total (N = 124), Full review indicated (N = 73), and Full review not indicated (N = 51). The rows include Median age (IQR), Sex (Male and Female percentages), Antimicrobial on survey date (Yes and No percentages), Surgery in the 30 days prior to or during admission (Yes and No percentages), Median length of stay (Days), and Ward type (Medical (non-ICU), Surgical (non-ICU), and ICU percentages). Notable trends include a higher percentage of males in the full review not indicated group and a higher percentage of patients on antimicrobials in the full review indicated group.

Navigate back to Table 1..

## Table 2. Long description

A table summarizing prescribed antimicrobials by WHO anatomical therapeutic chemical classification. The table has 2 columns and 22 rows. Column headers are Antimicrobial medication and Number of patients receiving (%). Row 1: Total patients on an antimicrobial, 79 (100). Row 2: Piperacillin and beta-lactamase inhibitor, 26 (32.9). Row 3: Amoxicillin and beta-lactamase inhibitor, 2 (2.5). Row 4: Meropenem, 20 (25.3). Row 5: Cefazolin, 12 (15.2). Row 6: Ceftriaxone, 9 (11.4). Row 7: Cefepime, 6 (7.6). Row 8: Ceftazidime and beta-lactamase inhibitor, 2 (2.5). Row 9: Aztreonam, 1 (1.3). Row 10: Sulfamethoxazole and trimethoprim, 3 (3.8). Row 11: Amikacin, 1 (1.3). Row 12: Ciprofloxacin, 7 (8.9). Row 13: Levofloxacin, 3 (3.8). Row 14: Vancomycin, 30 (38.0). Row 15: Metronidazole, 6 (7.6). Row 16: Colistin, 4 (5.1). Row 17: Isavuconazole, 3 (3.8). Row 18: Micafungin, 3 (3.8). Row 19: Voriconazole, 3 (3.8). Row 20: Amphotericin B, 2 (2.5). Row 21: Fluconazole, 2 (2.5). Row 22: Acyclovir, 3 (3.8).

Navigate back to Table 2..

## Table 3. Long description

A table with three columns and six rows detailing healthcare-associated infections (HAI) by patient, relevant device presence, and ward. The columns are labeled HAI, Relevant device, and Ward. The rows list specific infections, device presence, and corresponding wards for each patient. Row 1: Patient A, Bloodstream infection, Yes, Surgical. Row 2: Patient B, Bloodstream infection, No, Medical. Row 3: Patient B, Pneumonia, No, Medical. Row 4: Patient C, Pneumonia, Yes, ICU. Row 5: Patient D, Pneumonia, Yes, ICU. Row 6: Patient D, Urinary tract infection, Yes, ICU. Row 7: Patient E, Surgical Site Infection, N/A, Medical/Surgical. Row 8: Patient F, Surgical Site Infection, N/A, Surgical.

Navigate back to Table 3..
