# Supplementary material for: Pilot implementation of the GAIHN point prevalence survey on healthcare-associated infections at a Jordanian tertiary oncology hospital, an international partnership with the U.S. Centers for Disease Control and Prevention
Source: Antimicrob Steward Healthc Epidemiol. 2026 Sep 23;6(1):e290. doi: 10.1017/ash.2026.10859 (PMC13760432; doi:10.1017/ash.2026.10859)
Supplement: Supplementary file 1 [file S2732494X26108596sup001.docx]

Case Definitions and Classification

**Healthcare-associated infection (HAI) Definition**

**Pneumonia**

Any patient on an antimicrobial (route=ANY, indication=infection OR unknown) **AND** any of the following present on the survey date or the 14 days prior:

- mechanical ventilation or intubation
- chest imaging ordered
- sputum/lower respiratory tract culture ordered

**OR** clinical impression of pneumonia noted from chart/care team.

- Symptomatic evidence:
  - at least one of the following symptoms present on event date (or the day prior)
    - fever >38°C
    - leukocytosis (>12,000 WBC/mm^3^) OR leukopenia (<4000 WBC/mm^3^)

AND

- at least one of the following symptoms present on event date (or the day prior) that were not present on admission date (hospitalization DAY 1) or hospitalization DAY 2
  - new onset of purulent sputum, or change in character of sputum (color, odor, quantity, consistency);
  - cough or dyspnea or tachypnea (respiration rate >25 breaths per minute);
  - suggestive auscultation: rales, crackles, bronchial breath sounds, rhonchi, wheezing;
  - worsening gas exchange (e.g., oxygen desaturation or increased oxygen requirements or increased ventilation demand)
- Radiographic evidence (collected within 7 days of event date): One or more serial chest X-rays or CT-scans with new and persistent OR progressive and persistent infiltrate, consolidation, or cavitation.
- Confirmed Microbiologic Evidence (collected within 7 days of event date):
  - Positive quantitative culture from lower respiratory tract specimen
    - broncho-alveolar lavage (BAL) with a threshold of >10^4^ CFU^2^/ml or ≥5 % of BAL obtained cells contain intracellular bacteria on direct microscopic exam (classified on the diagnostic category BAL);
    - protected brush or distal protected aspirate with a threshold of >10^3^ CFU/ml;
    - quantitative culture of lower respiratory tract specimen (e.g., tracheal or endotracheal aspirate) with a threshold of 10^6^ CFU/ml
    - Any of the above lower respiratory tract specimens with a semi-quantitative result of 2+, 3+, or 4+ or “moderate”, “heavy”, “many” or “numerous”, growth
- Suspected Microbiologic Evidence (collected within 7 days of event date):
  - positive growth in culture of pleural fluid;
  - positive exams for pneumonia with virus or particular germs (virus, *Bordetella*, *Legionella*, *Chlamydia*, *Aspergillus*, mycobacteria, mycoplasma, *Pneumocystis carinii*):
    - positive detection of viral antigen or antibody from respiratory secretions (e.g., EIA, FAMA, shell vial assay, PCR);
    - positive direct exam or positive culture for any of the above organisms from bronchial secretions or tissue;
    - Detection of antigens in urine (*Legionella*).
  - Positive (as defined by the laboratory) sputum culture or non-quantitative lower respiratory tract specimen culture.
- Device risk factor: Intubation present in the 2 days prior to event date (not including devices inserted on event date)

**Urinary Tract Infection**

UTI-A: Symptomatic UTI with confirmatory microbiology

- Patient has one of the following signs or symptoms on event date (or the day prior) that were not present on admission date (hospitalization DAY 1) or hospitalization DAY 2:
  - fever (>38°C)
  - urgency
  - frequency
  - dysuria
  - suprapubic tenderness: whether elicited by palpation or provided as a subjective complaint of suprapubic, bladder or pelvic pain or discomfort

AND

- patient has a positive urine culture (≥ 10^5^ microorganisms per ml of urine with no more than two species of microorganisms) collected within 7 days of event date

UTI-B: Symptomatic UTI without confirmatory microbiology

- Patient has one of the following signs or symptoms on event date (or the day prior) that were not present on admission date (hospitalization DAY 1) or hospitalization DAY 2:
  - fever (>38°C)
  - urgency
  - frequency
  - dysuria
  - suprapubic tenderness: whether elicited by palpation or provided as a subjective complaint of suprapubic, bladder or pelvic pain or discomfort

AND

- at least one of the following collected within 7 days of event date:
  - positive dipstick for leukocyte esterase and/or nitrites;
  - pyuria (urine specimen with ≥ 10 WBC/ml or ≥ 3 WBC/high-power field of unspun urine) or other positive result as defined by the facility laboratory
  - organisms seen on Gram stain of unspun urine;
  - other positive urine culture as defined by the facility laboratory

**Bloodstream Infection**

BSI-A: Blood stream infection with confirmatory microbiology

- One positive blood culture for a recognized^[1]^ pathogen collected within 7 days of event date

BSI-B: Symptomatic blood stream infection with microbiology

- Patient has at least one of the following signs or symptoms on event date (or the day prior):
  - fever (>38°C)
  - chills
  - hypotension

AND one positive blood culture for a common skin contaminant collected within 7 days of event date.

**Surgical Site Infection**

Any patient that underwent a surgery during the current admission **OR** underwent a surgery at the surveyed facility in the 30 days prior to admission date

Symptom Evidence:

- Purulent drainage with or without laboratory confirmation, from the incision.
- One of the following combination of signs or symptoms of infection:
  - fever AND pain or tenderness localized to incision site, OR
  - fever AND swelling, redness, or heat localized to incision site
- An abscess or other evidence of infection involving the incision or organ/space found on direct examination, or during reoperation.

Microbiologic Evidence:

- Organisms isolated from an aseptically obtained culture of fluid or tissue from the incision or abscess involving the incision or organ/space

**Superficial:** The infection involves only skin and subcutaneous tissue of the incision. There should be NO mention of involvement of fascia, muscle, bone, or organs.
*Examples of terms used to describe a superficial SSI include cellulitis, dermatitis, panniculitis, and subcutaneous abscess.*

**Deep incisional**: The infection involves deep soft tissue (e.g., fascia, muscle) of the incision. There should be NO mention of involvement of any bones, organs, or organ spaces.
*Examples of terms used to describe a deep incisional infection include fasciitis, myositis, and intramuscular abscess.*

**Organ/space:** The infection involves any part of the anatomy other than the incision (e.g., organs and spaces) which was opened or manipulated during an operation.
*Examples of terms used to describe an organ/space infection include any abscess within a body organ, meningitis, ventriculitis, osteomyelitis, and peritonitis.*
